# Catheterization of the Air-Passages

**Published:** 1855-08

**Authors:** 


*Catheterization of the Air-Passages.*—This controversy is assuming a formidable shape. The committee appointed by the New York Academy of Medicine, to consider the paper presented by Dr. Horace Green, some six months since, have reported. The majority report is signed by Drs. Willard Parker, John O. Stone, J. T. Metcalf, and Isaac Wood, in full. Dr. Alex. H. Stevens sent in an individual report endorsing the majority; Dr. James Anderson assented to the majority report so far as it referred to the practicability of the operation, but thought that the therapeutical question had not received that investigation which would warrant the committee in pronouncing upon it; and finally, Dr. B. F. Barker submits a minority report, sustaining Dr. Green, and of course differing widely from the majority.

Neither report has as yet been acted upon, and we propose to furnish our readers with a *resumé* of the affair as it now stands, leaving them to form their own conclusions as to who is right or who wrong. When the matter has been decided, we may, or may not, make it a subject of criticism.

Dr. Horace Green read before the N. Y. Academy of Medicine, at its meeting in December, 1854, a paper on "Diseases of the Air-Passages," and advocated the injection of a solution of nitrate of silver into the lungs, by means of gum elastic tubes passed down into the trachea and bronchi; furthermore asserting that such practice was feasible in the hands of the expert surgeon, that the catheter may be at will passed into either bronchus, and that no great inconvenience resulted to the patient from the presence of th s foreign body in the air-passages.

The gentlemen whom we have named were appointed "to inquire into and examine" the proposed treatment. They report that they decided in the outset:

"1. To settle the question of the passage of the instrument into the air-passages; and

"2. To ascertain, as far as possible, the utility of injections of nitrate of silver into these passages."

Under the first head they consider the *History* of the operation, the *Results of Experiments*, and the *facility* with which the operation may be performed.

*History.* Hippocrates first recommended the operation—as he did all manner of impossible things about which he knew nothing. Dessault, Ryland, and others, mention and approve it.

*Results of Experiments.* Thirty-eight patients were experimented on. In eleven cases the catheter was carried into the trachea. This fact they considered proved, and only proved, by the symptoms manifested by the patients, the opinion of the operator being unreliable when he trusts to his own senses and disregards the symptoms.

"The symptoms which distinguish the passage of an instrument into the trachea and œsophagus, may be thus contrasted:

| *Trachea.* | *Œsophagus.* |
|---|---|
| 1st. Suffusion of the face rapidly increasing to turgescence and lividity. | 1st. Suffusion of the face slightly, rapidly subsiding, with cessation of retching and cough. |
| 2d. Great anxiety and alarm, not easily pacified. | 2d. Little anxiety; easily pacified. |
| 3d. Eyes wild, staring, and overflowing with tears. | 3d. Eyes natural, slight suffusion from tears. |
| 4th. Cough violent and spasmodic. | 4th. Little or no cough. |
| 5th. Respiration greatly disturbed; inspiration loud, hoarse, and stridulous; expiration attended with violent cough, like that of laryngeal phthisis, ejection of bronchial mucus through the tube, and finally free breathing through the tube. | 5th. Respiration little if at all disturbed. |
| 6th. Voice extinguished; a hoarse whisper interpreted with difficulty. | 6th. Voice distinct, often quite natural. |
| 7th. Retching slight." | 7th. Retching and vomiting, a common symptom." |

These rational signs the committee regard as the surest signs of the success of the operation. In several instances where Dr. Green supposed the tube to be in the trachea, (the œsophageal signs being present) he was found to be in error, as was proven by the vomiting of contents of the stomach through the tube. The committee, therefore, assume that the tube was never in the trachea, except in those cases where the tracheal signs were present.

As to the *facility* of the operation we cut a condensed report from the

N. Y. Medical Times, premising that Dr. Green had insisted upon the previous preparation of the patient as a condition of success:

"The facility of the operation does not depend upon the previous preparation of the patient, as insisted upon by Dr. Green; for, with the exception of Mesmore,patients who had frequent applications of the caustic to the fauces for six months, were equally intolerant as the new patients.

"Much depends upon the character and form of the instruments. With a tube curved by a stylet to a form corresponding to a circle of six inches diameter, in thirteen cases, five failed, or thirty-eight per cent. of failure, which proves that the trachea may be entered with a very considerable/certainty.

"With a tube with a small curve, such as used by Dr. Green, in thirty eight cases, thirty-five failed, or ninety-two per cent. of failure. With the sponge probang, in eighteen cases, there were eighteen failures, or 100 per cent. of failure.

"The conclusion is, that the sponge probang, or slightly curved tube, cannot be made to enter the trachea; but, if largely curved, it can; that local application within the trachea is difficult, and rarely successful; and whether an instrument may be passed at will into the right or left bronchi, the committee leave to the academy to decide from these facts.

"The committee considered themselves excused from entering into the therapeutical value of the application, as proposed, on the grounds that they do not think it advisable to discuss a mere theoretical question. A post mortem of a consumptive is given, where death followed in twenty-six hours after the operation."

The statement that the "committee considered themselves excused from entering into the therapeutical value of the operation," is an error on the part of the Times, the remark on which it is based being confined in the report to the practicability of injecting *tubercular cavities*.

The evidence before the committee on this department of their investigation was, however, very slender. They confine themselves to the mention of two cases which did not come under their observation at any time after the injection, and to a detailed history of the fatal case mentioned. This case occurred at Bellevue Hospital, and the death was unmistakably the result of the operation. The committee conclude:

"That in the great majority of instances where injections are supposed to have been thrown into the lung, through a tube, they have passed directly into the stomach.

"That as regards the utility of injections of nitrate of silver into the lungs, the facts thus far developed, in the experiments of your committee, lead them to regard the operation as one fraught with danger, as well as difficulty."

—— Thus much for the majority report. The minority report was made by our friend Prof. B. F. Barker, and we shall pursue the same plan with it that we have done with the majority document, except that we shall confine our abstract to points raised in our synopsis of Prof. Willard Parker's statement. We do this for the sake of brevity and fairness, believing that we have stated the leading issues.

*History.* Dr. Barker gives an elaborate sketch of the history of the operation, much more complete in its details than the other.

*Results of Experiments.* Dr. Barker denies that the rational signs relied on by the committee are conclusive as to the location of the tube. He quotes many instances from various writers where the suffocative symptoms were not experienced, and claims that the larynx having been previously "educated" by the application of the nitrate of silver to the fauces and the parts about the larynx, such symptoms will not occur. He asserts, therefore, "*that the absence of the 'rational signs,' as given by the committee, is no evidence that the tube is not in the trachea, and that when the committee have decided on these grounds, that an experiment was unsuccessful, their decision was wholly unreliable.*"

*Facility of the Operation.* Dr. Barker claims that in order to give a fair trial to it, the operation should be performed after Dr. Green's method, and not without the preparation he claims to be necessary, or by an instrument of different form. On this ground he would throw out the cases at Bellevue Hospital, as they had not been properly prepared, and as according to the statement of the majority, they were frightened and uncontrollable. By some different method of calculation, Dr. Barker throws out a part of the cases as reckoned by the majority, and says that "Twenty-two patients were subjected to the experiment with the tube." * * * The experiments failed in nine patients, were successful in nine, and the results were doubtful in four," making fifty per cent. of success where the results were decided. Of the remainder of the thirty-eight cases, a part are set aside, for various reasons, until only ten are left, of which three were unsuccessful, three successful, and four doubtful, thus maintaining the average of success. Recurring to the reasons which induced him to reject the standard of success (suffocative symptoms) assumed by the majority, he claims a probability that these doubtful cases were really successful.

Relative to the sponge probang, Dr. Barker gives the measurement of the chordæ vocales, recapitulates the frequent instances in which large bodies have entered the trachea, refers to the opinions of many physicians, and finally assumes that it did not come within the province of the commiitee.

*Therapeutic value.* Dr. B. says that the committee reported upon the limited experience to be derived from these cases, while an abundant field of observation was open for them at Dr. Green's office. Relative to the fatal case, he says that Dr. Green neither advised, suggested, or performed the injection.

Here we leave the facts with our readers, with a promise to keep them posted as to the decision of the academy.

